# Occupational Exposure to Mercury at an Electronics Waste and Lamp Recycling Facility — Ohio, 2023

**DOI:** 10.15585/mmwr.mm7401a2

**Published:** 2025-01-09

**Authors:** Dallas S. Shi, Melissa Charles, Catherine Beaucham, Sheldon Walker, Walter Alarcon, Scott E. Brueck, Sophia K. Chiu, Nicholas Somerville

**Affiliations:** ^1^Division of Field Studies and Engineering, National Institute for Occupational Safety and Health, CDC; ^2^Epidemic Intelligence Service, CDC.

**Summary**

**What is already known about this topic?**

Workers in electronics waste and lamp recycling facilities face health risks from inhaling mercury vapor and mercury-containing dust.

**What is added by this report?**

At an Ohio electronics waste and lamp recycling facility, mercury vapor was found throughout, and six of 14 workers had elevated urine mercury levels. Among those with elevated urine mercury, the median job tenure was 8 months; four workers did not speak English, and five reported signs and symptoms consistent with mercury toxicity.

**What are the implications for public health practice?**

Employers at electronics waste and lamp recycling facilities are encouraged to evaluate mercury exposure and implement controls such as enhancing ventilation systems and providing training tailored to the worker.

## Abstract

Workers in electronics waste and lamp recycling facilities are at risk of exposure to elemental mercury through inhalation of mercury vapor and mercury-containing dust. Employers at an electronics waste and lamp recycling facility in Ohio that crushes mercury-containing lamps expressed concerns about mercury exposure from work processes and requested a health hazard evaluation by CDC’s National Institute for Occupational Safety and Health (NIOSH). In April 2023, NIOSH conducted a multidisciplinary investigation to assess elemental and inorganic mercury exposures, including epidemiologic, environmental, and ventilation assessments. Results indicated that mercury vapor was detected throughout the facility, with six of 14 workers having elevated urine mercury levels. These workers had a median job tenure of 8 months; four did not speak English, and five reported symptoms consistent with mercury toxicity, such as metallic or bitter taste, difficulty thinking, and changes in personality. Recommendations included improving the ventilation system, changing work practices to reduce mercury exposure, and providing training and communication tailored to the worker. As the electronic waste recycling industry continues to grow, it is important for employers to evaluate mercury exposure and safeguard employees using a hierarchy of controls. Health departments should consider monitoring occupational mercury exposure in recycling facilities, and clinicians should be aware of the potential for mercury toxicity among workers in these settings.

## Investigation and Results

Mercury exposure is an occupational hazard with serious health consequences, including neurological symptoms such as tremors, memory loss, and difficulty concentrating, as well as kidney damage and other systemic effects (*1*). Elemental mercury exposure occurs primarily through inhalation of mercury vapor, which can be rapidly absorbed into the bloodstream. Chronic exposure, even at low levels, can lead to cumulative health effects over time (*1*,*2*).

Occupational limits have been established to safeguard workers against mercury exposure. These limits include the American Conference of Governmental Industrial Hygienists (ACGIH) threshold limit value (TLV) of 25 *μ*g/m^3^, the National Institute for Occupational Safety and Health’s (NIOSH) recommended exposure limit (REL) of 50 *μ*g/m^3^, and the Occupational Safety and Health Administration’s (OSHA’s) permissible exposure limit (PEL) of 100 *μ*g/m^3^. ACGIH TLV and NIOSH REL are recommended exposure limits to prevent adverse health effects among workers; OSHA PEL is a legally enforceable limit.

Workers in electronics waste and lamp recycling facilities face unique risks for mercury exposure due to the crushing and processing of mercury-containing lamps (*3*). Mercury vapor and dust can become airborne, creating significant inhalation risks. In response to concerns raised by employers at an electronics waste and lamp recycling facility in Ohio about mercury exposure from work processes, NIOSH conducted a health hazard evaluation (HHE).* The evaluation, carried out in April 2023, involved a multidisciplinary team of industrial hygienists, epidemiologists, and medical officers. During a 2-day site visit, CDC investigators conducted a cross-sectional epidemiologic study by interviewing 15 workers, performed environmental sampling for mercury vapor, assessed the facility's ventilation system to identify potential sources and levels of mercury exposure, and offered spot urine testing (*4*). This activity was reviewed by CDC, deemed not research, and conducted consistent with applicable federal law and CDC policy.^†^

### Facility and Work Process Description

The facility was a two-story warehouse divided into four sections: 1) administrative areas; 2) common spaces (entrance, hallways, bathrooms, breakroom, conference room, locker room, and personal protective equipment [PPE] storage); 3) lamp recycling areas (lamp room, glass roll-off, shaker, and retort furnace); and 4) additional workspaces (material storage, battery and ballast sorting, and bulb storage). During an 8-hour work day, lamp room workers load mercury-containing bulbs onto a conveyor for crushing. A sorting machine divides the bulbs into glass (deposited in the glass roll-off area), metal, and mercury dust (further sieved into ultrafine dust by the shaker). The retort furnace, which extracts mercury from ultrafine dust using heat, was not in use at the time of HHE. Workers in the battery and ballast areas prepare electrode components, such as metal or graphite parts, for shipment to facilities where they are reused or recycled into new batteries or other products. Employees in the lamp room and retort furnace area wear half-mask elastomeric respirators (reusable respirators made from a flexible material that provides a tight seal and are equipped with replaceable cartridges for filtering mercury vapor), steel-toed boots, safety glasses, and a company-issued long-sleeved shirt.

### Worker Interviews and Spot Urine Testing

All 15 workers at the facility participated in a semistructured interview about employment history, work characteristics, signs and symptoms consistent with mercury toxicity, and medical and social histories. Workers were given the option to undergo spot urine testing for inorganic and elemental mercury at the time of the interview. Spot urine testing was chosen because of its convenience, instead of 24-hour urine or end-of-shift collection at the end of the workweek. Urine specimens were analyzed by Associated Regional and University Pathologists, Inc. (https://www.aruplab.com/) laboratories using inductively coupled plasma mass spectrometry, an analytic technique that can detect the concentration of elements and their isotopes in a sample. Creatinine levels, a marker of kidney function, were measured, and urine mercury-to-creatinine ratios were calculated for comparison with the ACGIH Biologic Exposure Index (BEI) of 20.0 *μ*g/g creatinine. BEI is a guideline value indicating the level of a substance in biologic samples below which most workers are unlikely to experience adverse health effects.

### Environmental and Personal Air Sampling Methodology

Direct area air sampling for elemental mercury vapor was conducted during 2 work days using a Jerome J405 atomic fluorescence mercury vapor analyzer (https://www.pine-environmental.com/products/jerome_j405). A total of 171 direct area air samples were measured at breathing height (approximately 5 ft [1.5 m] above floor level) to assess mercury vapor levels across the facility. Comparisons to occupational exposure limits were used to identify potential areas of concern within the facility. In addition, all workers were offered the opportunity to participate in personal air sampling, which involved collection of full-shift personal breathing zone samples for mercury vapor analysis during 2 days to directly compare against occupational exposure limits.

### PPE Use

Inconsistent use of recommended PPE was observed throughout the facility. Observations during the site visit revealed that, particularly in the lamp room where respirators are mandatory, workers frequently did not adhere to proper PPE use. Instances included employees removing their respirators or wearing them incorrectly, such as one employee using an N95 respirator with one of the straps cut off, severely compromising the respirator’s seal. Other observations included sporadic use of gloves and protective clothing. These observations were further corroborated by worker interviews. Some workers reported challenges with the fit and comfort of their PPE, while others cited a lack of understanding regarding the proper use and maintenance of equipment. Language barriers among workers appeared to exacerbate these issues, as training and communication were not always provided in workers’ preferred languages.

### Environmental Air Sampling Findings

Mercury was detected in all 171 direct area air samples (Figure). In areas outside of the lamp recycling areas (lamp room, glass roll-off, shaker, and retort areas), referred to as nonproduction areas, the median mercury vapor concentrations in the conference room (26.0 *μ*g/m^3^; range = 12.8–29.8 *μ*g/m^3^) and material storage area (60.5 *μ*g/m^3^; range = 10.1–89.7 *μ*g/m^3^) exceeded the ACGIH TLV of 25 *μ*g/m^3^. The median mercury vapor concentration in the material storage area also exceeded the NIOSH REL of 50 *μ*g/m^3^. In production areas, the median mercury vapor concentrations in the lamp room (35.8 *μ*g/m^3^; range = 2.5–91.1 *μ*g/m^3^), glass roll-off area (29.1 *μ*g/m^3^; range = 7.8–106.3 *μ*g/m^3^), and retort furnace area (26.1 *μ*g/m^3^; range = 10.9–67.5 *μ*g/m^3^) were also above ACGIH TLV. One sample from the glass roll-off area (106.3 *μ*g/m^3^) exceeded both NIOSH REL and OSHA PEL.

**FIGURE:**
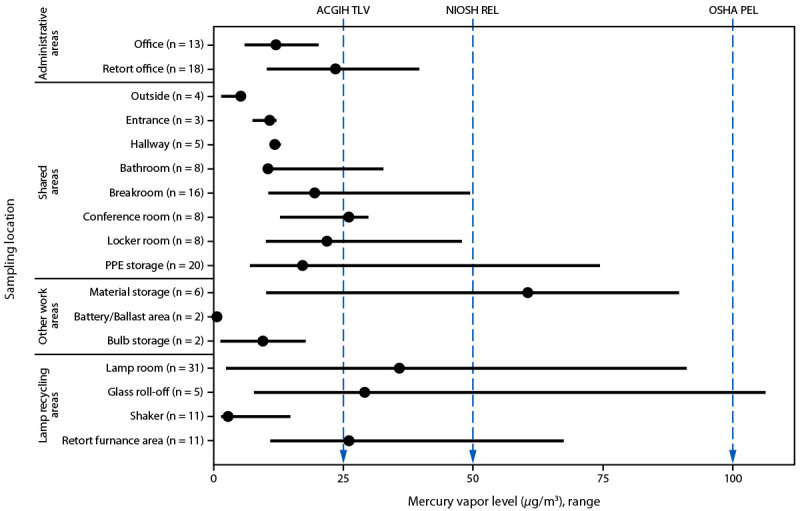
Median mercury vapor levels, by work location at an electronic waste and lamp recycling facility — Ohio, 2023 **Abbreviations:** ACGIH = American Conference of Governmental Industrial Hygienists; NIOSH = National Institute for Occupational Safety and Health; OSHA = Occupational Safety and Health Administration; PEL = permissible exposure limit; PPE = personal protective equipment; REL = recommended exposure limit; TLV = threshold limit value.

### Results of Urine Testing and Personal Air Sampling

All 15 employees participated in urine collection. One urine sample was too diluted to interpret. Among six workers in the lamp recycling area, the median mercury-to-creatinine ratio was 41.3 *μ*g/g, and the levels of five of these workers exceeded ACGIH BEI (Table 1). Among three workers in administrative areas and five in other work areas, the median urine mercury-to-creatinine ratios were 8.6 *μ*g/g and 5.8 *μ*g/g, respectively. Overall, six of 14 workers had spot urine mercury levels above ACGIH BEI, including five of six workers in the lamp recycling areas and one of five workers in other work areas. All six workers in the lamp recycling areas and three of those in other work areas participated in personal air sampling. Five of six workers in the lamp recycling areas had personal air exposures to mercury vapor above the ACGIH TLV of 25 *μ*g/m^3^ (median = 64.8 *μ*g/m^3^).

**TABLE 1:** Median spot urine mercury levels and personal mercury vapor exposure levels among workers at an electronic waste and lamp recycling facility, by primary work location (N = 15) — Ohio, 2023

Primary job location	No. of workers	Median (range) urine mercury to creatinine ratio (*μ*g/g)	No. (%) of samples >ACGIH BEI*	No. of personal air samples	Median (range) personal mercury vapor exposure (*μ*g/m^3^)^†^	No. (%) of samples >ACGIH TLV^§^
Lamp recycling areas	6	41.3 (16.1–64.0)	5 (83)	12	64.8 (10.7–81.8)	10 (83)
Administrative areas	3	8.6 (4.2–13.0)	0 (—)	0	—	—
Other work areas	5^¶^	5.8 (1.3–45.2)	1 (20)	6	6.6 (2.9–11.5)	0 (—)
**Total**	14**	**51.0 (1.3–64.0)**	**6 (43)**	**18**	**33.6 (2.9–81.8)**	**10 (56)**

**Abbreviations:** ACGIH = American Conference of Governmental Industrial Hygienists; BEI = biologic exposure index; TLV = threshold limit value.

* ACGIH BEI for inorganic mercury in urine is 20 *μ*g/g creatinine.

^†^ Personal air sampling was collected over the course of two shifts per worker. In total, nine workers participated with a total of 18 samples collected. Workers in the administrative areas did not participate in personal air sampling.

^§^ ACGIH TLV for elemental mercury is 25 *μ*g/m^3^.

^¶^ All five workers participated in urine testing; three participated in personal air sampling.

** Urine specimen from one employee was too diluted to interpret.

### Characteristics of Workers with Elevated Spot Urine Mercury Levels

Of the 14 workers whose spot urine samples were sufficiently concentrated for interpretation of mercury levels, six had levels exceeding ACGIH BEI (Table 2). Among these, all were male and four were Spanish-speaking. All eight workers with mercury levels below BEI primarily spoke English and worked in production areas. Median job tenure of workers with mercury levels above BEI was 8 months compared with 23 months among workers with mercury levels below BEI. Five of the six workers with levels above BEI reported signs and symptoms consistent with mercury exposure, including a metallic or bitter taste, difficulty thinking, or personality changes (three each); difficulty writing or loss of balance, light headedness, or dizziness (two each); and skin rash, headache, numbness or tingling in hands or feet, weight loss, or diarrhea (one each). (Participants could identify any signs or symptoms that began after their employment began at the recycling facility, and multiple signs and symptoms could be reported by each participant.) Four of the eight workers with levels below BEI reported no symptoms.

**TABLE 2:** Demographic characteristics and symptoms of electronic waste and lamp recycling facility workers with spot urine mercury levels above and below the American Conference of Governmental Industrial Hygienists biologic exposure index* (N = 14) — Ohio, 2023

Characteristic	No. (%), by urine mercury level	
	≤20 *μ*g/g creatinine	>20 *μ*g/g creatinine
**No. of workers**	8	6
**Median age, yrs (range)**	40 (25–53)	41 (35–54)
**Sex**		
Female	2 (25)	0 (—)
Male	6 (75)	6 (100)
**Primary language**		
English	8 (100)	2 (33)
Spanish	0 (—)	4 (67)
**Job tenure, mos, median (range)**	23 (14–144)	8 (3–32)
**Self-reported signs and symptoms^†^**		
Any sign or symptom	4 (50)	5 (83)
Metallic or bitter taste	1 (13)	3 (50)
Difficulty thinking	0 (—)	3 (50)
Changes in personality	0 (—)	3 (50)
Difficulty writing	0 (—)	2 (33)
Loss of balance, lightheadedness, or dizziness	0 (—)	2 (33)
Skin rash or sore	1 (13)	1 (17)
Headaches	3 (38)	1 (17)
Numbness or tingling in hands or feet	1 (13)	1 (17)
Unplanned weight loss	1 (13)	1 (17)
Diarrhea	1 (13)	1 (17)
No reported sign or symptom	4 (50)	1 (17)

* 20 *μ*g/g creatinine.

^†^ Reported signs and symptoms are not mutually exclusive. Participants could identify any symptoms that began after their employment began at the recycling facility, and multiple symptoms could be reported by each participant.

## Public Health Response

Recommendations to protect workers based on a hierarchy of controls^§^ approach were provided to the facility (*4*). Recommended engineering controls included installing local exhaust ventilation over the conveyer in the lamp room and maintenance of the facility’s heating, ventilation, and air conditioning systems. Other recommendations included implementing a workflow progressing from clean to dirty zones to prevent the spread of mercury to clean areas, improving housekeeping, tailoring training in workers’ preferred languages, and standardizing use of recommended PPE.

## Discussion

The expansion of the recycling industry offers opportunities to promote sustainable waste management practices but also raises challenges related to workers’ health (*5*). This investigation highlights occupational health concerns at an electronics waste and lamp recycling facility, where identification of environmental mercury vapor and individual worker urine mercury concentrations surpassing ACGIH safety thresholds indicate a need for enhanced protective measures and monitoring. Previous studies have consistently demonstrated the occupational hazards posed by mercury exposure in recycling and manufacturing settings, and underscore the importance of comprehensive safety protocols that help worksites adhere to recommended exposure limits (*3*,*6*). Observed inconsistent proper PPE use likely contributed to high urine mercury measurements despite the use of respiratory protection, indicating a need for enforcement of safety protocols and targeted training to support proper PPE use.

Elevated mercury vapor levels were also identified in areas of the facility not directly involved in lamp recycling. Although personal exposure measurements for mercury in these areas did not surpass ACGIH TLV, one worker with no direct involvement in lamp recycling had elevated urine mercury levels. This finding suggests that contamination of nonproduction areas can affect nonproduction workers. Mercury exposure below established occupational limits can have harmful health effects over time, including neurologic symptoms such as tremors, memory problems, and difficulty concentrating, as well as kidney damage (*1*,*2*). To mitigate these risks, comprehensive controls are essential. The diverse nature of recycling operations means that workers, regardless of their direct involvement with recycling processes, might be exposed to hazardous substances such as mercury.

This investigation identified a disparity in exposure levels among workers with different primary languages and job tenure, suggesting potential barriers to effective communication and training (*2*,*7*). These findings align with broader occupational health literature, which identifies language barriers and job tenure as factors influencing health and safety (*7*–*9*). The higher prevalence of self-reported symptoms among workers with elevated mercury levels reinforces the need for ongoing health monitoring to mitigate the adverse health effects of mercury.

Employers at recycling facilities can implement comprehensive exposure mitigation strategies that align with the hierarchy of controls. These strategies include enclosing spaces with the highest potential for mercury exposure to prevent contamination of nonproduction areas, improved ventilation, use of appropriate PPE, regular exposure surveillance, and training programs tailored to worker needs. Health departments with recycling facilities in their jurisdiction should be aware of the potential for mercury exposure, while clinicians should remain vigilant for signs and sympoms of mercury toxicity among workers in these environments. Regular monitoring is essential to ensure that controls are effective and to detect any changes in exposure levels (*10*).

## Footnotes

*
https://www.cdc.gov/niosh/hhe/default.html

† 45 C.F.R. part 46.102(l)(2), 21 C.F.R. part 56; 42 U.S.C. Sect. 241(d); 5 U.S.C. Sect. 552a; 44 U.S.C. Sect. 3501 et seq.

§ The hierarchy of controls is a framework that groups corrective actions by their likely effectiveness in reducing or removing hazards from the workplace. Levels in the hierarchy include elimination, substitution, engineering controls, administrative or work-practice controls, and PPE. https://www.cdc.gov/niosh/hierarchy-of-controls/about/index.html
